# A real-world study of the efficacy of a simplified pill count-based strategy for treating HCV/HIV coinfection patients

**DOI:** 10.1186/s12879-026-13895-2

**Published:** 2026-06-29

**Authors:** Bianchuan Cao, Ying Peng, Mei Liu, Ping Ding, Lingyu Tang, Mingxian Guo, Ling Liu, Cunliang Deng, Gang Wu, Li Zhong, Yongsheng Zou

**Affiliations:** 1 Department of Infectious Diseases, The Affiliated Hospital of Southwest Medical University, No.25 Taiping Street, Luzhou, 646000 China; 2 Department of Tuberculosis, The Affiliated Hospital of Southwest Medical University, Luzhou, 646000 China; 3 Infection and Immune Laboratory, The Affiliated Hospital of Southwest Medical University, Luzhou, 646000 China; 4 Antiviral Therapy Center, The First People’s Hospital of Yuexi County, Liangshan, 616650 China

**Keywords:** HCV/HIV coinfection, Direct-acting antiviral, Single-tablet regimen, Multi-tablet regimen, Resource-limited area

## Abstract

**Background:**

Hepatitis C virus (HCV) and human immunodeficiency virus (HIV) infections are major public health issues throughout the world. In resource-limited areas, successful treatment of HCV/HIV coinfection frequently faces challenges, particularly restricted access to drugs and poor treatment compliance. However, data comparing single-tablet regimen (STR) versus multi-tablet regimen (MTR) direct-acting antiviral (DAA) strategies, specifically focusing on pill burden, remain scarce in such settings.

**Methods:**

This single-center, retrospective cohort study enrolled patients with HCV/HIV coinfection between April 2023 and October 2025. All patients received DAA therapy in addition to the antiretroviral therapy (ART). Participants were divided into DAA STR (one pill daily) and DAA MTR (multiple pills daily) groups. The primary endpoint was sustained virologic response at least 12 weeks after completion of treatment (SVR).

**Results:**

A total of 105 patients with HCV/HIV coinfection were enrolled. The overall SVR rate was 96.2% (101/105). The DAA STR group had a numerically higher SVR rate than the DAA MTR group (98.8% vs. 88.0%, *P* = 0.041); however, this comparison was confounded by genotype. In a sensitivity analysis limited to non-genotype 3b patients (*n* = 87), SVR rate was 98.8% in the DAA STR group and 85.7% in the DAA MTR group (*P* = 0.155). The DAA STR demonstrated a numerical advantage in achieving SVR in all subgroups stratified by sex, age, HCV infection route, baseline HCV RNA level, HCV genotype, and ART regimen. Univariate logistic regression identified DAA regimen was the only factor associated with SVR (OR 10.773, 95% CI 1.067–108.743); however, because only 4 participants failed to achieve SVR, this analysis was exploratory and the estimate was statistically unstable.

**Conclusions:**

In this real-world study, both DAA STR and DAA MTR achieved high overall SVR rates in HCV/HIV coinfection in a resource-limited setting. The simplified DAA STR was associated with a numerically higher SVR, but the comparison was confounded by the selective allocation of genotype 3b patients to DAA MTR. The observed difference between DAA STR and DAA MTR may reflect treatment selection practices and genotype distribution rather than an independent effect of regimen simplification. The simplified pill count-based strategy remains a practical option, but its advantage over MTR was confounded by genotype selection in this cohort. Our findings support the feasibility of both approaches and highlight the need for large, prospective, genotype-balanced comparative studies with integrated adherence monitoring.

## Background

Hepatitis C virus (HCV) and human immunodeficiency virus (HIV) infections are major public health issues throughout the world [1–4]. These two viruses have similar transmission routes, resulting in an increased prevalence of HCV/HIV coinfection, particularly in high-risk populations, such as intravenous drug users and individuals engaging in unprotected sexual practices [5, 6]. Liangshan, China, has a high prevalence of HCV/HIV coinfection due to past disease epidemics and social factors [7–9]. Coinfection by both viruses exacerbates the progression of liver disease, increasing the risk of liver fibrosis, cirrhosis, and liver cancer, and adversely affecting patients’ outcomes [10–12]. Therefore, effective antiviral therapy is crucial for improving prognosis. In recent years, the use of direct-acting antiviral (DAA) strategies has significantly improved the success of HCV treatment, while antiretroviral therapy (ART) has transformed HIV infection into a chronic, manageable disease [13–16]. Nevertheless, in resource-limited areas, successful treatment of HCV/HIV coinfection frequently faces challenges, particularly restricted access to drugs and poor treatment compliance, as well as drug interactions, thereby severely affecting therapeutic efficacy [8, 17].

The pill count burden has a significant effect on treatment adherence [18, 19]. Indeed, a greater number of daily tablets is associated with higher risks of missed doses, incorrect administration, or even treatment discontinuation [20, 21]. The simplification of treatment regimen is a key strategy for enhancing medication compliance. Simplified pill count-based strategy typically involves reducing the daily number of tablets required, such as switching multi-tablet regimen (MTR) to single-tablet regimen (STR). In terms of ART, STR has been found to significantly improve treatment compliance among HIV patients [22–24]. Several studies from China have confirmed high SVR rates with various DAA regimens in HCV/HIV coinfection. Nevertheless, the comparative effectiveness of a simplified single-tablet versus multi-tablet approach, specifically examining pill burden reduction as a strategy in a resource-limited setting, has not been directly evaluated. In this study, the efficacy of a simplified pill count-based antiviral strategy for HCV/HIV coinfection was evaluated using real-world data to optimize treatment strategies for HCV/HIV coinfection.

## Methods

### Study participants

This was a single-center, retrospective cohort study conducted at the Antiviral Therapy Center of the First People’s Hospital of Yuexi County between April 2023 and October 2025. We screened patients with HCV/HIV coinfection who received ART and DAA. The inclusion criteria were: (1) Age ≥ 18 years; (2) Confirmed diagnosis of HCV/HIV coinfection; (3) Completion of DAA-anti-HCV treatment on the basis of ART with a minimum 12-week follow-up. The exclusion criteria were: (1) Comorbid liver cirrhosis or liver cancer; (2) Previous treatment with DAA; (3) Pregnant or lactating women; (4) Discontinuation of treatment or loss to follow-up; (5) Incomplete baseline clinical records.

### Methods

All patients received DAA-anti-HCV treatment based on ART according to clinical guidelines, drug accessibility, and individual circumstances. The DAA and ART regimens both involved STR and MTR. The DAA STR was defined as requiring only one tablet daily for anti-HCV treatment, such as Sofosbuvir/Velpatasvir (SOF/VEL), Elbasvir/Grazoprevir (EBR/GZR). The DAA MTR was defined as requiring multiple tablets daily for anti-HCV treatment, such as SOF/VEL plus Ribavirin (RBV), Coblopasvir + Sofosbuvir (CLP + SOF), Emitasvir + Sofosbuvir. The ART STR was defined as requiring only one tablet daily for anti-HIV treatment, such as Bictegravir Sodium, Emtricitabine and Tenofovir Alafenamide Fumarate (BIC/FTC/TAF). The ART MTR required multiple tablets daily for anti-HIV treatment, such as Tenofovir Disoproxil Fumarate (TDF) + Lamivudine (3TC) + Lopinavir/Ritonavir (LPV/r) or Zidovudine (AZT) + 3TC + LPV/r.

### Data collection

Both demographic (age, sex) and clinical (ART regimen, HBV coinfection, HCV infection route, baseline HCV RNA level, HCV genotype and DAA regimen) information were recorded. Safety data were collected via review of medical records and patient interviews; laboratory monitoring included complete blood count, liver and renal function. Adverse events (AEs) were graded according to CTCAE v5.0.

### Endpoint

The primary endpoint was the proportion of patients who achieved a sustained virologic response (SVR), defined as an HCV RNA level lower than the limit of detection at least 12 weeks after completion of the anti-HCV treatment.

### Statistical analysis

Data processing and statistical analysis were performed using SPSS 26.0. Normally distributed measurement data were expressed as mean and standard deviation ($\:\stackrel{-}{x}$±SD), and were compared using independent samples t-tests. Ordinal data were compared using Mann-Whitney U tests. Categorical data are presented as numbers (percentages) and were analyzed using the χ² test or Fisher’s exact test. Univariate logistic regression analysis was employed to identify variables associated with the achievement of SVR. All tests were two-tailed, with *P* < 0.05 indicating statistical significance.

## Results

### Baseline characteristics

**Fig. 1:**
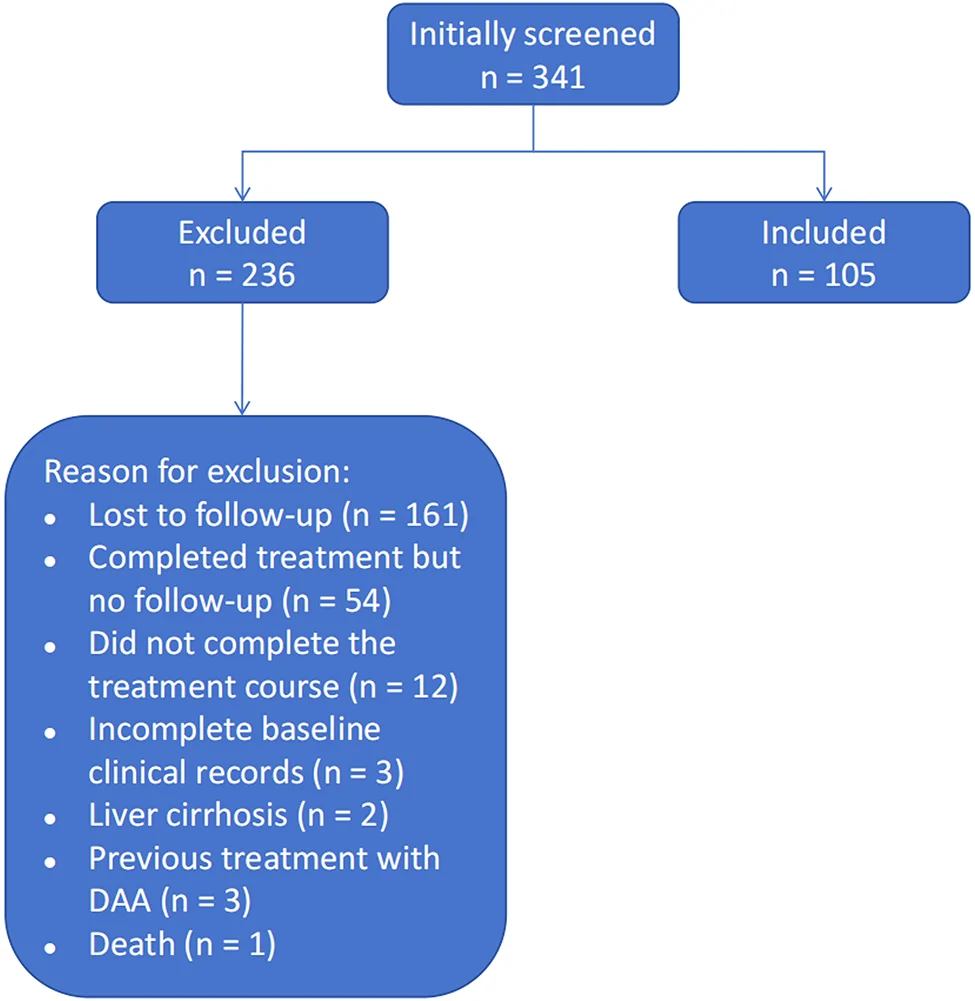
Patient selection flowchart

A total of 341 patients were initially screened. Of these, 161 were lost to follow-up, 54 completed treatment but did not complete follow-up, 12 did not complete the treatment course, 3 had incomplete baseline clinical records, 2 had liver cirrhosis, 3 had previous treatment with DAA, and 1 died. Thus, 105 patients were included (Fig. 1). Excluded patients did not differ significantly in age or sex from included patients, although a full comparison was limited by missing data. Among the 105 participants, 75 were male, and 30 were female. The mean age was 40.1 ± 6.1 years old. The predominant route of HCV infection was intravenous drug use (64.8%). The HCV RNA levels ranged predominantly between 1 × 10^5^ and 1 × 10^6^ IU/mL (31.4%). The HCV genotype 1b was the most common (36.2%). The DAA regimen was predominantly associated with STR (76.2%), while the ART regimen was mostly MTR (90.5%). Apart from the difference in the HCV genotype, no statistically significant differences were found between the DAA STR and DAA MTR groups in terms of sex, age, HBV coinfection, HCV infection route, baseline HCV RNA level, or ART regimen. Table 1 shows the baseline characteristics of the participants.

**Table 1:** Baseline characteristics

Subgroups	Total (*n* = 105)	DAA STR (*n* = 80)	DAA MTR (*n* = 25)	*P*-value
**Sex**				0.943
Male	75 (71.4%)	57 (71.3%)	18 (72.0%)	
Female	30 (28.6%)	23 (28.7%)	7 (28.0%)	
**Age**,** year**	40.1 ± 6.1	40.8 ± 5.9	38.1 ± 6.5	0.052
**HBV coinfection**	2 (1.9%)	2 (2.5%)	0 (0%)	1.000
**HCV infection route**				0.296
Heterosexual contact	37 (35.2%)	26 (32.5%)	11 (44.0%)	
Intravenous drug use	68 (64.8%)	54 (67.5%)	14 (56.0%)	
**Baseline HCV RNA (IU/mL)**				0.965
< 1 × 10^6^	55 (52.4%)	42 (52.5%)	13 (52.0%)	
≥ 1 × 10^6^	50 (47.6%)	38 (47.5%)	12 (48.0%)	
**HCV genotype**				< 0.001
3b	18 (17.1%)	0 (0%)	18 (72.0%)	
Non-3b	87 (82.9%)	80 (100%)	7 (28.0%)	
**ART regimen**				0.064
ART STR	10 (9.5%)	10 (12.5%)	0 (0%)	
ART MTR	95 (90.5%)	70 (87.5%)	25 (100%)	

ART, antiretroviral therapy; DAA, direct-acting antiviral; HBV, hepatitis B virus; HCV, hepatitis C virus; MTR, multi-tablet regimen; RNA, ribonucleic acid; STR, single-tablet regimen

### Overall efficacy and sensitivity analysis

**Fig. 2:**
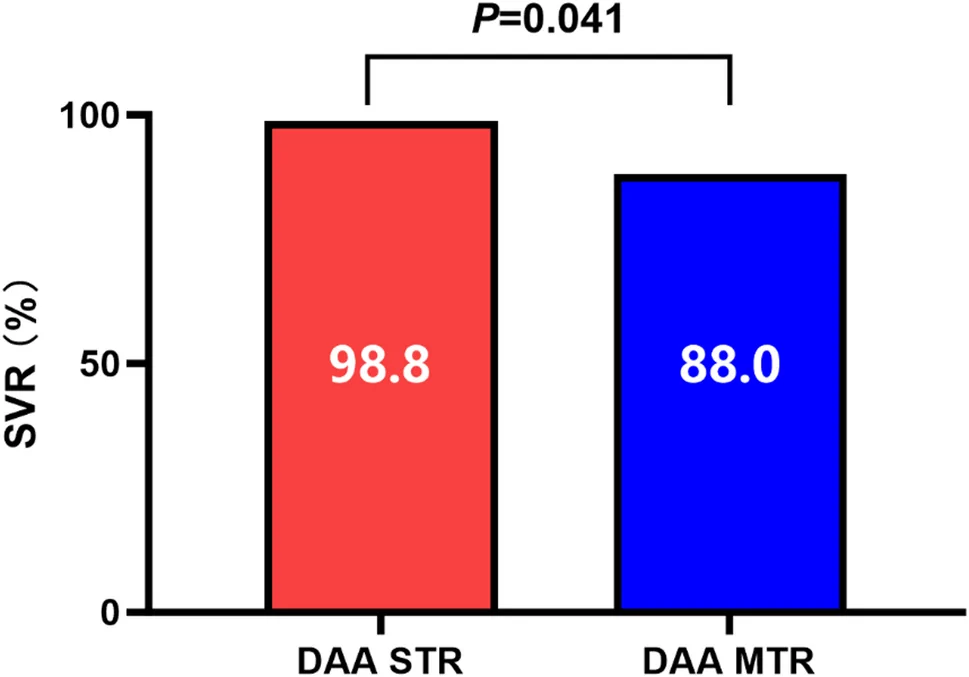
Overall efficacy of DAA in HCV/HIV Coinfection

One hundred and one patients achieved SVR, representing an overall SVR rate of 96.2% (101/105). The DAA STR group had a numerically higher SVR rate (98.8%, 79/80) than the DAA MTR group (88.0%, 22/25) (*P* = 0.041) (Fig. 2). However, genotype distribution was imbalanced: all genotype 3b patients (*n* = 18) were in the DAA MTR group. To address this confounding, we performed a sensitivity analysis restricted to non-3b genotypes (*n* = 87). In this subset, the SVR rates were 98.8% (79/80) in the DAA STR group and 85.7% (6/7) in the DAA MTR group (*P* = 0.155), indicating that the overall difference lost statistical significance after removing the genotype effect. These results highlight that the STR advantage is largely attributable to the concentration of difficult-to-treat genotype 3b in the DAA MTR group.

### Subgroup analysis

The DAA STR regimen demonstrated a numerical advantage over the DAA MTR regimen in achieving SVR in all subgroups stratified by sex, age, HCV infection route, baseline HCV RNA level, HCV genotype, and ART regimen. Table 2 shows the efficacy analysis by subgroups. Due to the small sample size in the DAA MTR group (*n* = 25), changes of a single patient can markedly alter percentages; subgroup comparisons are for hypothesis generation only.

**Table 2:** Efficacy analysis by subgroups

Subgroups	SVR (%)		*P*-value
	DAA STR	DAA MTR	
**Sex**			
Male	98.2% (56/57)	83.3% (15/18)	0.041
Female	100% (23/23)	100% (7/7)	NA
**Age**,** year**			
< 39	100% (32/32)	92.3% (12/13)	0.289
≥ 39	97.9% (47/48)	83.3% (10/12)	0.099
**HCV infection route**			
Heterosexual contact	100% (26/26)	90.9% (10/11)	0.297
Intravenous drug use	98.1% (53/54)	85.7% (12/14)	0.105
**Baseline HCV RNA (IU/mL)**			
< 1 × 10^6^	100% (42/42)	92.3% (12/13)	0.236
≥ 1 × 10^6^	97.4% (37/38)	83.3% (10/12)	0.139
**HCV genotype**			
3b	-	88.9% (16/18)	NA
Non-3b	98.8% (79/80)	85.7% (6/7)	0.155
**ART regimen**			
ART STR	100% (10/10)	-	NA
ART MTR	98.6% (69/70)	88.0% (22/25)	0.055

NA indicates that the statistical test was not applicable due to zero events in one subgroup

ART, antiretroviral therapy; DAA, direct-acting antiviral; HCV, hepatitis C virus; MTR, multi-tablet regimen; RNA, ribonucleic acid; STR, single-tablet regimen; SVR, sustained virologic response

### Factors associated with SVR achievement

Univariate logistic regression was used to analyze the 105 study participants, using SVR achievement as the dependent variable and sex, age, HCV infection route, baseline HCV RNA level, HCV genotype, ART regimen, and DAA regimen as independent variables. Table 3 shows the univariate logistic regression analysis for achieving SVR. Univariate logistic regression identified DAA regimen was the only factor associated with SVR (OR 10.773, 95% CI 1.067–108.743). However, this association was completely confounded by genotype because all genotype 3b patients received DAA MTR. Given the small number of SVR failures (*n* = 4), this analysis is exploratory and the confidence interval is wide; the result should be interpreted with caution because of statistical instability. Due to the small number of events (4 failures) and the collinearity, multivariable adjustment was not feasible and would be unreliable. Thus, we present only unadjusted estimates.

**Table 3:** Univariate logistic regression analysis for achieving SVR

Factors	SVR Achievement	*P*-value	OR (95% CI)
**Sex**		0.998	-
Male	94.7% (71/75)		
Female	100% (30/30)		
**Age (years)**		0.474	2.316 (0.233–23.031)
< 39	97.8% (44/45)		
≥ 39	95.0% (57/60)		
**HCV infection route**		0.665	1.662 (0.167–16.564)
Heterosexual contact	97.3% (36/37)		
Intravenous drug use	95.6% (65/68)		
**Baseline HCV RNA (IU/mL)**		0.291	3.447 (0.347–34.266)
< 1 × 10^6^	98.2% (54/55)		
≥ 1 × 10^6^	94.0% (47/50)		
**HCV genotype**			
3b	88.9% (16/18)	0.107	0.188 (0.025–1.435)
Non-3b	97.7% (85/87)		
**ART regimen**		0.999	-
ART STR	100% (10/10)		
ART MTR	95.8% (91/95)		
**DAA regimen**		0.044	10.773 (1.067-108.743)
DAA STR	98.8% (79/80)		
DAA MTR	88.0% (22/25)		

ART, antiretroviral therapy; CI, confidence interval; DAA, direct-acting antiviral; HCV, hepatitis C virus; MTR, multi-tablet regimen; OR, odds ratio; RNA, ribonucleic acid; STR, single-tablet regimen; SVR, sustained virologic response

### Analysis of SVR failure

Four of the 105 participants failed to achieve SVR. These patients had received ART via MTR. Among the four patients, three were treated with the DAA MTR regimen, three had baseline HCV RNA levels exceeding 6 × 10⁶ IU/mL, and two had HCV genotype 3b. Table 4 shows the analysis of SVR failure.

**Table 4:** Analysis of SVR failure

Patient No.	ART regimen	HCV infection route	Baseline HCV RNA (IU/mL)	HCV genotype	DAA regimen
2	TDF+3TC + LPV/r	Intravenous drug use	1.54 × 10^7^	Untyped	SOF/VEL
9	TDF+3TC + LPV/r	Intravenous drug use	6.2 × 10^6^	3b	SOF/VEL + RBV
28	TDF+3TC + LPV/r	Heterosexual contact	6.5 × 10^6^	1b	CLP + SOF
97	TDF+3TC + LPV/r	Intravenous drug use	2.47 × 10^5^	3b	SOF/VEL + RBV

3TC, lamivudine; ART, antiretroviral therapy; CLP, coblopasvir; DAA, direct-acting antiviral; HCV, hepatitis C virus; LPV/r, lopinavir/ritonavir; RBV, ribavirin; RNA, ribonucleic acid; SOF, sofosbuvir; TDF, tenofovir disoproxil fumarate; VEL, velpatasvir

### Safety analysis

Safety was assessed in all treated patients. No grade ≥ 3 adverse events or treatment discontinuations due to adverse events occurred, and no treatment regimens were changed or interrupted due to drug-related adverse events. In the DAA MTR group, four patients reported mild fatigue or headache; all events were self-limiting. Laboratory monitoring did not reveal any clinically significant hepatic or renal toxicity.

## Discussion

Since the launching of the second phase of its prevention and control measures for the control of major infectious diseases, including AIDS, in 2021, by Liangshan Prefecture, the scope of the program has expanded beyond AIDS to include other major infectious diseases, such as hepatitis C. However, due to limitations in available medical resources, the achievement of optimal treatment efficacy under these conditions has become an urgent issue requiring resolution. In terms of the goal of the World Health Organization (WHO) to eliminate HCV infection globally by 2030, the 2022 WHO HCV guidelines emphasize the application of “simplified strategies” for HCV diagnosis and treatment delivery [25]. Our study aimed to evaluate the efficacy of simplified pill count-based strategy for antiviral therapy in cases of HCV/HIV coinfection in resource-limited areas. The findings provide practical guidance for regions faced with restricted resources and the management of patients with multiple infections.

The results demonstrated an overall SVR rate of 96.2% for HCV/HIV coinfection, consistent with previous reports on the efficacy of DAA in real-world settings [26, 27]. The SVR rate was 98.8% in patients who received DAA STR, in contrast to a rate of 88.0% in those receiving DAA MTR, representing a statistically significant difference (*P* = 0.041). These findings suggest that the real-world efficacy of DAA STR for HCV/HIV coinfection is associated with a higher SVR, but this association is confounded by genotype distribution. Any suggestion that the observed difference in SVR might be attributable to improved adherence or treatment compliance associated with STR remains hypothetical, because adherence was not directly measured. The simplified pill count-based strategy, by reducing the daily number of tablets, has potential for improving both treatment compliance and persistence [28]. Despite the lower SVR rate for DAA MTR compared to DAA STR, this regimen remains an indispensable treatment option due to its relative flexibility in terms of drug combinations and drug accessibility. The SVR rate of DAA MTR is relatively low in this cohort, partly due to the presence of genotype 3b, which is difficult to treat with DAA [29–31].

In this study, most patients with HCV/HIV coinfection received the DAA STR (76.2%), reflecting the growing preference for simplified regimens in clinical practice. Compared with the DAA MTR, the DAA STR avoids the complexity of multi-drug combinations and reduces the medication burden, making it particularly suitable for patients with HCV/HIV coinfection who require long-term ART. Furthermore, regardless of sex, age, HCV infection route, baseline HCV RNA level, HCV genotype, or ART regimen, patients with HCV/HIV coinfection who received the DAA STR demonstrated numerically higher SVR rates than those receiving the DAA MTR. This suggests that the advantages of DAA STR are also applicable to patients with HCV/HIV coinfection and contribute to better treatment adherence. Although this treatment could be compatible with better adherence, adherence was not assessed, so such an explanation remains speculative. Additionally, although only 9.5% of the patients in this study received ART STR, all patients receiving ART STR achieved SVR, indicating that the simplification of ART regimens based on pill count is associated with successful HCV antiviral therapy. For patients in the Liangshan region, where levels of education, socioeconomic status, and disease awareness are generally low, complex medication regimens are more likely to lead to missed doses, incorrect administration, or even treatment discontinuation [32, 33]. The DAA STR provides maximal simplification of the medication process, thereby reducing the burden on the patient–a factor that may theoretically improve treatment compliance, although this was not directly evaluated. The results of the logistic regression analysis revealed that the DAA STR was more likely to lead to SVR than the DAA MTR. Nevertheless, all patients infected with the HCV genotype 3b received DAA MTR. This genotype is difficult to treat, and clinical management therefore favors intensified treatment. This aligns with current guideline-recommended treatment strategies [30]. Although this study did not observe a statistically significant association between HCV genotype and SVR achievement, this may have resulted from the relatively small sample size and potential bias in terms of genotypic distribution. The striking imbalance of genotype 3b, which was exclusively treated with DAA MTR, suggests that clinicians consciously selected intensified MTR for difficult-to-treat patients. Indeed, when the analysis was restricted to non-3b patients, the SVR gap narrowed and became non-significant, implying that genotype distribution, rather than pill count per se, may be the primary driver of the observed outcome difference.

Among the four subjects who did not achieve SVR, three had baseline HCV RNA levels exceeding 6 × 10⁶ IU/mL, suggesting that high HCV RNA at baseline may negatively impact the outcomes of HCV antiviral therapy. In addition, two of the four non-SVR subjects had HCV genotype 3b, suggesting the need for enhanced clinical management for this difficult-to-treat genotype.

A substantial proportion of initially screened patients were excluded because of loss to follow-up (161/341, 47.2%) or incomplete follow-up (54/341, 15.8%). These excluded patients may differ systematically from those included in the analysis. For instance, patients who were lost to follow-up might have had lower adherence, which could affect SVR rates. Consequently, the reported SVR rate in our study may overestimate the true efficacy in the source population. The generalizability of our findings is therefore limited to patients who complete treatment and follow-up, and the results should be interpreted with awareness of this potential selection bias.

Our study has some limitations. First, its single-center, retrospective design with a small sample size, particularly in the DAA MTR group (*n* = 25), limits generalizability and statistical power. Second, non-randomized treatment allocation led to indication bias, as all genotype 3b patients were exclusively treated with DAA MTR, thereby confounding the regimen comparison. Third, the high proportion of excluded patients due to loss to follow-up or incomplete follow-up may have introduced selection bias, affecting the external validity of the SVR estimates. Fourth, this was a retrospective review in a resource-limited setting. In this region, ART has been highly popularized, certain follow-up parameters such as CD4^+^T cell count, HIV RNA level, liver fibrosis status, and liver and renal function are not routinely retested as part of standard infectious disease care at the primary level, leading to unavoidable unmeasured confounding. These crucial potential confounders were not systematically recorded, preventing adequate adjustment. Fifth, adherence was not directly measured in this study. Consequently, any adherence-related interpretations remain hypothetical. Sixth, the low number of SVR failures (*n* = 4) precluded reliable multivariable modeling; furthermore, the complete collinearity between HCV genotype 3b and DAA MTR prevented the simultaneous inclusion of genotype and regimen in any adjusted analysis. Large, prospective, genotype-balanced trials with integrated adherence monitoring are warranted.

## Conclusion

In this real-world study, both DAA STR and DAA MTR achieved high overall SVR rates in HCV/HIV coinfection in a resource-limited setting. The simplified DAA STR was associated with a numerically higher SVR, but the comparison was confounded by the selective allocation of genotype 3b patients to DAA MTR. The observed difference between DAA STR and DAA MTR may reflect treatment selection practices and genotype distribution rather than an independent effect of regimen simplification. The simplified pill count-based strategy remains a practical option, but its advantage over MTR was confounded by genotype selection in this cohort. Our findings support the feasibility of both approaches and highlight the need for large, prospective, genotype-balanced comparative studies with integrated adherence monitoring.

## Data Availability

The data and materials associated with this article will be available to external researchers interested in collaboration upon the submission and approval of a data analysis plan. Requests for data should be submitted to yongshengz@swmu.edu.cn.
